# Supplementary material for: A set of multi-entry identification keys to African frugivorous flies (Diptera, Tephritidae)
Source: Zookeys. 2014 Jul 24;(428):97–108. doi: 10.3897/zookeys.428.7366 (PMC4143993; doi:10.3897/zookeys.428.7366)
Supplement: Supplementary material 6 — Key to Ceratitis [file zookeys-428-097-s006.zip › SF6_ZooKeys_key to Ceratitis/key/SF6_ZooKeys_key to Ceratitis/Media/Html/Ceratitis flava.htm]

Ceratitis (Pterandrus) flava, n


***Ceratitis*** **(*Pterandrus*) *flava* De Meyer & Freidberg**

Body
length: 5.43 (5.30-5.55) mm; wing length: 5.82 (5.55-6.05) mm.

Male

Head: Antenna yellow. First flagellomere three times as long
as pedicel. Arista with short to moderately long rays; ventral rays shorter and
sparser than dorsal rays, especially basally. Frons flat to convex; yellow;
with short scattered setulae largely of same color as frons. Frontal setae well
developed. Face yellowish white, gena slightly darker yellow. Genal seta and
setulae dark, well developed.

Thorax: Postpronotal
lobe yellowish white, without spot. Mesonorum: ground color pale gray with
distinct yellowish orange tinge; with streaks and darker markings but without
distinct spots, prescutellar white markings separate. Scapular setae dark.
Scutellum yellowish white, basally without distinct spots, apically with three
separate dark spots, extending to apical 0.33. Subscutellum yellowish brown.
Anepisternum completely pale; setulae pale.

Legs:
Yellow; setation typical for subgenus, mainly pale. Foreleg: femur posteriorly
with dispersed short pale setulae; posterodorsal and posterior rows longer and
black; ventrally with row of long black setae. Midleg: femur ventrally with few
moderately long black setulae on distal 0.25,  distinct feathering; tibia with
black setulae dorsally along distal 0.8 and ventrally along distal half,
setulae forming row but without distinct feathering. Hindleg: femur at apical
0.25 with longer setulae dorsally and ventrally.

Wing:
bands yellowish brown. Interruption between marginal and discal bands near vein
R_1_ clear and complete; cubital band free; medial band absent;
crossvein R-M just proximal to middle of discal cell. Apex of vein R_1_
distal to level of crossvein R-M. Crossvein DM-Cu oblique anterobasally.

Abdomen:
Mostly yellow. Tergites 2 and 4 across posterior part with pale gray band,
weakly silvery when viewed from certain angle; tergite 3 occassionaly with very
weak interrupted brownish line across posterior margin. Male epandrium in
lateral view with lateral  surstylus elongate, posterior lobe elongate and
slightly curved.

Female

As
male except for the following characters: Legs without feathering; forefemur
posteroventrally with pale pilosity. Abdominal tergite 3 with small brown
transverse band along  posterior 0.25. Oviscape slightly longer than
preabdomen. Aculeus at least 10 times longer than wide; lateral margin very
weakly sinuous.

(Description
after De Meyer & Freidberg, 2006)
